# Multiscale model for the optimal design of pedestrian queues to mitigate infectious disease spread

**DOI:** 10.1371/journal.pone.0235891

**Published:** 2020-07-09

**Authors:** Pierrot Derjany, Sirish Namilae, Dahai Liu, Ashok Srinivasan

**Affiliations:** 1 Aerospace Engineering, Embry-Riddle Aeronautical University, Daytona Beach, Florida, United States of America; 2 College of Aviation, Embry-Riddle Aeronautical University, Daytona Beach, Florida, United States of America; 3 Computer Science, University of West Florida, Pensacola, Florida, United States of America; London School of Hygiene and Tropical Medicine, UNITED KINGDOM

## Abstract

There is direct evidence for the spread of infectious diseases such as influenza, SARS, measles, and norovirus in locations where large groups of people gather at high densities e.g. theme parks, airports, etc. The mixing of susceptible and infectious individuals in these high people density man-made environments involves pedestrian movement which is generally not taken into account in modeling studies of disease dynamics. We address this problem through a multiscale model that combines pedestrian dynamics with stochastic infection spread models. The pedestrian dynamics model is utilized to generate the trajectories of motion and contacts between infected and susceptible individuals. We incorporate this information into a stochastic infection dynamics model with infection probability and contact radius as primary inputs. This generic model is applicable for several directly transmitted diseases by varying the input parameters related to infectivity and transmission mechanisms. Through this multiscale framework, we estimate the aggregate numbers and probabilities of newly infected people for different winding queue configurations. We find that the queue configuration has a significant impact on disease spread for a range of infection radii and transmission probabilities. We quantify the effectiveness of wall separators in suppressing the disease spread compared to rope separators. Further, we find that configurations with short aisles lower the infection spread when rope separators are used.

## Introduction

Pedestrian crowds are commonly observed in all public locations offering entertainment, transportation, social or religious activities. The mass gathering of people congregated in limited space often elevates the risk of infectious disease spread due to the increased contacts between susceptible and infectious individuals. Further, individuals with different levels of vulnerability and receptivity due to variations in genetic background and intervention usage often congregate in touristic sites [1]. There is direct evidence for the occurrence of multiple epidemic outbreaks in high pedestrian density locations such as transportation hubs, entertainment venues, (e.g. theme parks, stadiums) and mass gatherings [2–13]. Gautret and Steffen [2] report that sixty-eight cited instances of outbreaks among crowds occurred between 1980 and 2016. Numerous reports deal with the spread of diseases like influenza, SARS, and measles during air travel [3–5]. Examples of epidemics in entertainment venues include the influenza outbreak in 2002 during the winter Olympiad [6] and the measles outbreak in Disney World in 2016 resulting in 125 cases [7]. Several outbreaks of directly transmitted gastrointestinal and respiratory diseases have been reported in religious and social outdoor mass gatherings [8–10], international meetings [11, 12] and concert halls [13].

Disease spread in high pedestrian density locations is inherently a multidisciplinary and multiscale problem involving epidemiology and crowd dynamics. Deterministic [14] and stochastic [15] epidemiological models including Susceptible-Infected-Recovered (SIR) models are effective tools for understanding epidemic spread. However, such models do not account for discrete human interactions in pedestrian crowds. Computationally intensive agent-based models *e*.*g*. EpiSimdemics [16], and stochastic models [17] include human interactions through behavioral rules but are targeted at modeling simple interactions over large populations and geographical areas [16, 17], rather than evaluating the impact of fine-scale interactions. Instances mentioned above involve a high density of pedestrians over relatively small areas. Modeling non-uniform mixing in such instances and designing strategies for mitigation can only be achieved through multiscale modeling involving the combination of epidemic modeling with pedestrian crowd dynamics.

Understanding pedestrian dynamics and efficient crowd management practices are essential to enable effective flow of pedestrians, and for meeting safety standards in high pedestrian density environments noted above. Pedestrian crowd management often involves the combination of crowd psychology [18] and engineering methods for assessing the capacities of corridors, ramps, stairs, and other bottlenecks [19]. While several approaches including cellular automata [20], fluid flow models [21] have been used for modeling pedestrian dynamics, social force models [22, 23] have the advantage of evaluating the complete individual trajectories necessary for contact estimation in epidemic studies. Since its conception, there have been several advances in social force models involving force field estimations [24], algorithmic developments [25, 26] and applications in situations like panic [27], traffic dynamics [28] and evacuation [29]. Namilae et al. [30, 31] have used pedestrian dynamics described by the social force model in a multiscale model to study the spread of epidemics during air travel.

Despite separate developments in pedestrian dynamics and epidemiology, there is a paucity of epidemiological models that utilize detailed information from pedestrian dynamics for contact estimation. There is a strong correlation between contact and infection rates in several disease epidemics such as SARS [32] and Ebola [33]. Given the preponderance of epidemic outbreaks in high pedestrian density locations, a model that accounts for pedestrian dynamics in contact estimation can be a design tool for developing mitigation strategies. In this paper, we develop such a multiscale model and utilize it to study disease spread in pedestrian queues. Winding queue formation is a ubiquitous crowd control procedure. Consequently, individuals in crowded gatherings often spend a significant amount of time in waiting queue lines. In the multiscale model, pedestrian dynamics are used to generate trajectories of pedestrian motion and estimate the rate of contact between infected and susceptible individuals. We incorporate this information into a stochastic infection dynamics model with infection transmission probability and contact radius as primary inputs. This generic model is applicable for several directly transmitted diseases like Ebola, SARS, and H1N1 influenza by varying the input parameters related to infection probabilities and transmission mechanisms. We utilize this multiscale model to analyze disease spread in various pedestrian queue configurations, suggest preferred layouts, and design strategies that would reduce contacts and consequently mitigate the overall disease spread.

## Modeling methodology

### Pedestrian dynamics

To first estimate the number of contacts between susceptible and infectious individuals, we model each mobile pedestrian as a particle and immobile objects like walls or barriers as groups of stationary particles. The evolution of pedestrian particles and their interaction with other pedestrians and stationary particles are described by molecular dynamics like the social force model [23]. The net force ${\bar{f}}_{i}$ acting on an *i*^*th*^ pedestrian (or particle) can be defined as:

$${\bar{f}}_{i}=\frac{m_{i}}{\tau }\left({\bar{v}}_{0}^{i}(t)-{\bar{v}}^{i}(t)\right)+\sum_{j\neq i}{\bar{f}}_{ij}(t)=m_{i}\frac{dv_{i}}{dt}$$ (1)

with the pedestrian position at a given time obtained by integration as ${\bar{r}}^{i}(t)=\int {\bar{v}}^{i}(t)dt$. Here ${\bar{v}}_{o}^{i}(t)$ refers to the desired velocity of pedestrian, and ${\bar{v}}_{}^{i}(t)$ is the actual velocity, *m*_*i*_ is the particle’s mass and *τ* is the time constant. The momentum generated by a pedestrian’s intention, denoted by $\frac{m_{i}}{\tau }({\bar{v}}_{0}^{i}(t)-{\bar{v}}^{i}(t))$, results in a self-propulsion force that is balanced by a repulsion force ${\bar{f}}_{ij}(t)$ to obstacles in the direction of motion. In this study, we use the Lennard–Jones type repulsion term used earlier by Namilae et al. [30, 31].

While Eq (1) describes the general motion of pedestrians, we need to introduce modifications to this equation to account for slow-moving pedestrian queues. Pedestrians in a queue move at the speed of the nearest person ahead in the line. To model this scenario, we introduce location dependence to the desired velocity in the self-propulsion term as:

$$\begin{array}{c} v_{0}^{i}(t){\overset{⌢}{e}}_{1}=\\ \left\{(v_{A}+\gamma_{i}v_{B}\right)\left(1-\frac{\delta }{\min\left\{r_{ij{|}_{front}};i\neq j\right\}}\right)\;{\overset{⌢}{e}}_{1};\;\delta =\{\begin{array}{c} \delta_{1};if\;i\&j\;of\;same\;group\\ \delta_{2};if\;i\&j\;of\;different\;groups \end{array}\\ 0;if\;r_{ij{|}_{front}}<\delta \end{array}$$ (2)

where ${\overset{⌢}{e}}_{1}$ is the desired direction of motion. *v*_*A*_ and *γ*_*i*_*v*_*B*_ are the deterministic and stochastic components of the desired velocity respectively. The values of walking speed terms (*v*_*A*_ and *γ*_*i*_*v*_*B*_) can be varied to obtain a given distribution of age groups and gender of travelers [34]. *δ* is the cut-off distance constant between the *i*^*th*^ and *j*^*th*^ pedestrians at which the desired velocity of the *i*^*th*^ pedestrian reduces to zero velocity. *δ* is the minimum distance up to which two pedestrians can come close before the rear pedestrian stops to prevent overlap with the front pedestrian.

To mimic the real-life scenarios, we also account for the formation of groups of pedestrians. The groups’ formation is controlled by adjusting the distance (*δ*) in Eq (2). Literature [35] indicates that pedestrian spacing is different between pedestrians belonging to a group (e.g. family or friends in the queue) and other pedestrians. An average distance of *δ*_1_ = 0.46 m is chosen for pedestrian particles within the same group, while this distance between independent pedestrians is given a value of *δ*_2_ = 0.64 m.

### Contact estimation and infection model

Consider a population of size N consisting of I(t) infected and S(t) susceptibles at time t. The pedestrian’s position (*r*_*i*_(*t*)) evolves through the pedestrian dynamics model and is a function of age, sex and infection status. A susceptible can become infected when coming into direct contact with an infected individual. Given the trajectory of pedestrians over time, the number of contacts *m*_*i*_ can be evaluated by counting the instances when the distance between the *i*^*th*^ and *j*^*th*^ pedestrians (*r*_*ij*_) is less than a virus-specific contact radius (x). This transmission distance (x) used to define the contact is dependent on the type of pathogen and mechanisms for its spread. For diseases like Ebola, studies indicate that the primary mode of transmission is through contact droplets [36, 37]. Consequently, a distance that enables direct touch needs to be used for estimating contact for such diseases. Other infectious diseases like SARS and influenza are known to be transmitted by both shorter and longer range airborne mechanisms [38, 39]. Studies show that micrometer-sized aerosol clouds generated during cough can travel over 2 m [40, 41]. We vary the contact radius between these distances to account for the various infection spread mechanisms. Duration of contact is also needed to define a contact. Here, a contact is defined when a susceptible pedestrian is in the proximity of infective pedestrian within the contact distance (x) for a period of 4 seconds. The contact duration is chosen based on the breathing cycle as time sufficient to complete one inhalation of a contagion laden particle [42, 43].

Next, consider the probability (*P*_*inf*_) that a contact between a susceptible and an infective results in a successful infection transmission. We can divide this input parameter into two components: A viral shedding probability distribution (P_c_) which is a function of time since acquiring infection for the specific virus in question, and a pathogen spread mechanism component (P_m_). This includes contributions of several independent mechanisms comprising (a) aerosol exposure and inhalation probability (P_a_) common in infections such as SARS and influenza [38, 39], (b) Coarse pathogen droplet inoculation (P_d_) common in infectious diseases like Ebola [36]. Other mechanisms including fomite mechanism, which involves contaminated surface-to-hand transfer would contribute to the infection spread, but such mechanisms do not involve human-to-human contacts in this context and are not considered here. The infection probability would then be defined as:

$$P_{\inf}=P_{c}.P_{m}=P_{c}\left(P_{a}+P_{d}\right)$$ (3)

**Fig 1:**
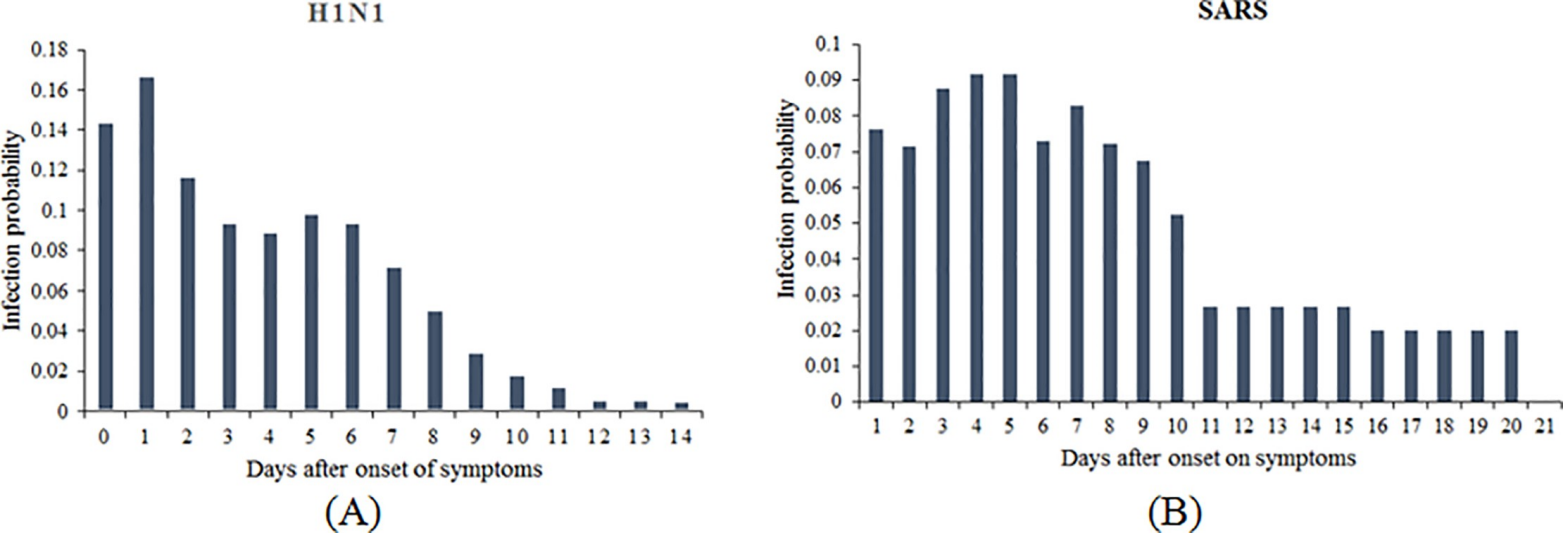
Viral shedding probability distributions (P_c_). (A) H1N1 influenza. (B) SARS virus.

First, consider the viral shedding probability distribution (P_c_). Studies indicate that the amount of viral shedding is typically dependent on the length of the incubation period and the number of days since the appearance of symptoms. In a previous study [31], we used CDC data on the amount of RNA (ribonucleic acid) virus copies in the blood serum since the illness contraction to generate this probability distribution for Ebola [44]. A similar approach can be used for other diseases, for example, for SARS pathogen, the viral gene expression of the nucleocapsid (N) protein, detected at different rates along with the evolution of the virus from post-onset of the symptoms till convalescence is indicative of viral shedding [45]. For influenza, nasal, oral or ocular shedding of the H1N1 virus has been detected by determining the relative equivalent unit from viral RNA level [46]. Such data can be used to generate the P_c_ distribution. Fig 1 shows the viral shedding distributions we generated based on [45] and [46] for SARS and H1N1 influenza respectively.

There are many formulations in the literature to compute the mechanism-specific probability of transmission. Table 1 lists the details of the popular mechanisms for aerosol and coarse droplet mechanisms. The functional form of the aerosol inhalation probability is described in the data-driven modeling framework in Teunis *et al*. [47], which in turn is based on Riley’s Dose-response model [48]. The probability for coarse droplet inoculation mechanism considers the droplet cone emitted during expiratory events like coughing [49].

**Table 1:** Formulations for generating mechanism-specific probability distributions.

Mechanism	Equations	Notes	References
Aerosols mechanism	$P_{a}=\left(1-e^{-\frac{QC_{a}\tau }{V_{o}}}\right)$	*Data-driven model framework based on dose-response model*	[47, 48]
		*C*_*a*_—maximum initial concentration of contagion in aerosol suspension	
		*τ*—exposure time	
		Q—respiration rate of susceptibles	
		*V*_*o*_—volume of infection envelope	
Coarse droplet inoculation	$P_{d}=\frac{S_{A}}{S_{C}}.\frac{V_{C}}{V_{o}}$	*Model based on expiratory droplet cone*	[49]
		*V*_*C*_—volume of cone in which droplet can fall	
		*V*_*o*_—room or exposure volume	
		*S*_*A*_—exposed mucosa surfaces	
		*S*_*C*_—circular area base of the cone	

The probability that an infectious individual “i” in the crowd comes into contact with other individuals is m_i_/N, where m_i_ is the number of contacts. Using Bayes’ theorem of conditional probability, *P(contact and infection) = P (infection |contact). P (contact)* = $P_{inf}.\frac{m_{i}}{N}$. To account for the demographic stochasticity of the susceptible individuals, the number of newly infected by this infective “i” is estimated by a binomial distribution I_i_ (t)~B(n_i_,p_i_) with parameters n_i_ = S_i_ (t-1), the number of susceptibles exposed to the contagion at time t, and ${p_{i}=P}_{inf}.\frac{m_{i}}{N}$. Eq (3) is used for estimating P_inf_.

For each infective individual, all the possible permutations are considered, i.e. the infective is considered to be in all possible positions in the queue. Binomial distributions are obtained to estimate a range of newly infected pedestrians with variations in the position and infectivity of the infective pedestrian. Denote by the variable *λ* the possible number of newly infected pedestrians ranging from zero to the maximum obtained number N_inf_ (*λ* = 0,…,*λ*_*i*_,…,N_inf_). The mean binomial distribution of the number of people infected at time t by all the possible permutations is computed using Eq (4) below. Here *Comb* denotes the number of combinations of infective positions and *w*_*i*_ is the frequency of obtaining *λ*_*i*_ newly infected in the computations. The day post-onset of symptoms which defines the infectivity (see Fig 1) is denoted by *c*. We combine the probability distributions and average them as given by:

$$I(t)∼\sum_{c=1}^{d}\sum_{i=1}^{i_{c}^{0}}\left\{\mathrm{Binomial}\;\left[S_{i}(t-1),P_{m}\cdot P_{c}\frac{m_{i}(t-1)}{N}\right]\right\}*\;w_{i}\left(\lambda_{i}\right)/\mathrm{Comb}$$ (4)

Note that the contacts are defined when pedestrians are within a specific transmission distance which is dependent on the transmission mechanism. Instead of using fixed parameters for defining contact, we will treat the contact distance and transmission prability as parameters in assessing epidemic spread. We will vary these parameters over a broad range to model the different scenarios (diseases and transmission mechanisms) for several pedestrian queue configurations. Expelled fine aerosols travel farther and remain suspended for a longer time than coarse droplets [40, 41]. We account for coarse droplets and aerosols transmission mechanisms by varying the contact radius parameter between 0.9 and 2.1 meters (36–84 inches). The contact radius range is based on the dynamics and the estimated travel distance of coarse droplets and fine aerosols expelled by an infective member from his respiratory tract during talking, breathing or coughing [40, 41]. We vary the transmittance probability *(P*_*inf*_*)* between 0.025 and 0.2 to account for the variation in the infectivity of different diseases. This range is considers the transmission probability calculations based on viral shedding [44–46] discussed earlier (Fig 1). The model parameters and the ranges are tabulated in Table 2.

**Table 2:** Numerical values and ranges of the parameters used in the multiscale model.

Parameter	Description	Estimated value/range
*v*_*A*_ + *γ*_*i*_*v*_*B*_	Pedestrian’s free speed	1.00–1.55 m/s
*γ*_*i*_	Random number	0–1
δ_1_	Cut-off distance between pedestrians of the same group	0.46 m
δ_2_	Cut-off distance between pedestrians of different groups	0.64 m
P_inf_	Infection probability	0.025–0.2
R	Contact radius	0.9–2.1 m

### Model application to pedestrian queue configurations

Pedestrian winding queues are an essential component of crowd management. These queues are often unidirectional and have different widths and configurations to fit the available area. The queues are often separated by rope stanchions for their ease of use. However, temporary walls could also be used for this purpose. Examples of such queues usage include airport security, waiting areas at theme parks and other crowded places. Within the same line and among adjacent lines, many susceptibles are often within the contact radius and viral infection may propagate if an infectious pedestrian is present.

**Fig 2:**
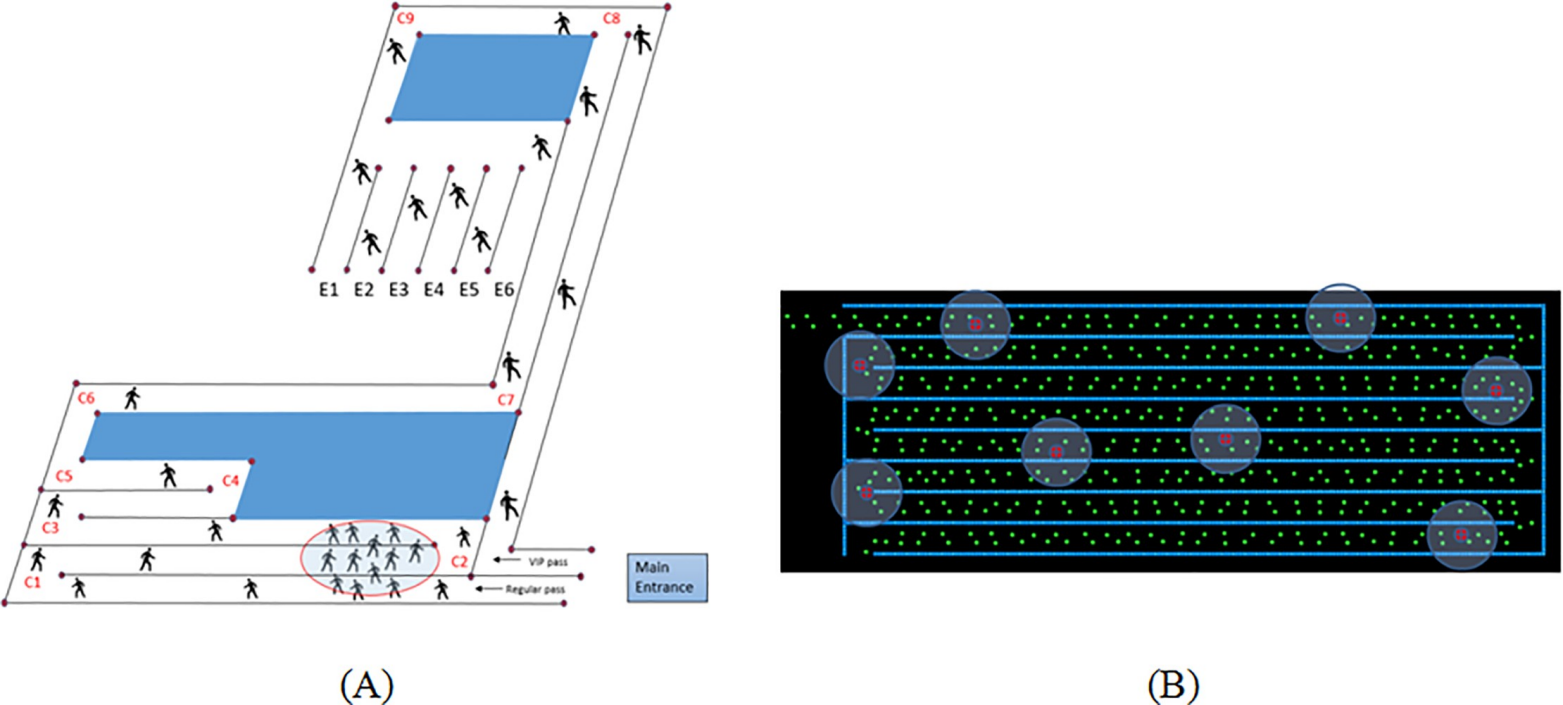
Replication of real life pedestrian motion in queues using simulations. (A) Schematic representation of an actual pedestrian queue in an entertainment venue. (B) Simulation snapshot of a corresponding model.

We evaluate the role of motion pattern and contact creation between neighboring pedestrians, for different queue configurations. The aisles’ geometry, orientation. number of inlets and exits are altered between the different configurations. To model queue configurations that are used in practice, we used the dimensions of a waiting queue similar to those in typical theme park attraction as shown in Fig 2. We used these dimensions as a basis for the different configurations modeled in the study. Social force based pedestrian models have been validated in numerous studies [50, 51]. The model described here has been shown to reproduce pedestrian evacuation data from airplanes as well as reproduce the experimentally verified speed-density diagrams [31, 52].

**Fig 3:**
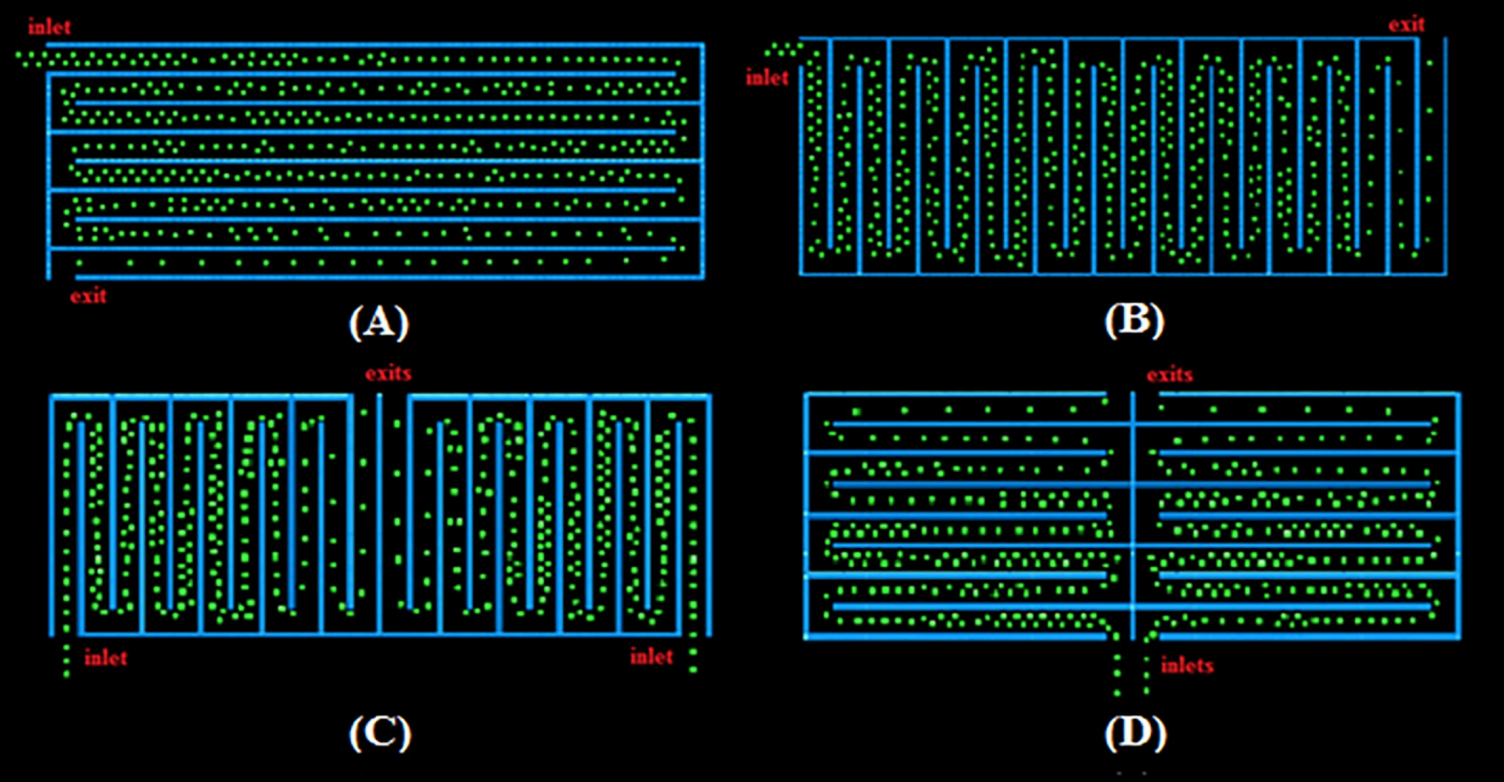
Evolution of pedestrians (t = 125s) from simulation of double queue rectangular layouts. (A) Configuration 1. (B) Configuration 2. (C) Configuration 3. (D) Configuration 4.

We utilize the queue layout shown in Fig 2 as the basis for evaluating the effect of the layout and shape of the queue configurations. The aisles’ length and orientation are altered between the configurations of the same area and aisle width. We investigated four different rectangular configurations with the same shape and area as shown in Fig 3. The four configurations are split vertically (configurations in Fig 3B and 3C) or horizontally (Fig 3A and 3D). Configurations in Fig 3A and 3B have one inlet and one exit whereas configurations in Fig 3C and 3D have two inlets and two exits due to the existence of separated zones. The width of the pedestrian lanes remains 1 m, which allows some pedestrians belonging to the same group to form a double line. The four configurations are termed Configurations 1, 2, 3 and 4 respectively.

We also investigate the relation between the layout shape and the contact evolution, by modeling four square floor plans of the same area as above configurations. In all the simulations, a total of 600 pedestrians are distributed within the waiting area. We take into account the possibility of pedestrians within a group walking side-by-side. In general, presence of groups would reduce the average distance between pedestrians and increase the pedestrian density, therefore would increase the number of contacts and the number of new infections. For all of the configurations, the number of contacts between pedestrians is calculated where rope separators or temporary walls are placed between the aisles. For rope separators, contact extends to pedestrians in the neighboring aisles, whereas for temporary walls, transmission due to contact is limited only between the pedestrians within the same aisle. The data of pedestrian contact is then combined with the infection model to estimate infectious disease spread. We consider the situation of a single infective in the queue. The infectious individual is unidentifiable; his/her rank in the queue is not known *apriori*. Therefore, all permutations of the infectious individual’s position are simulated to determine the average number of contacts for a given queue configuration. This results in 600 combination for six variations of each configuration. We then parametrize the contact radius and transmission probability and analyze if and how the queue layout and features impact the disease spread.

**Fig 4:**
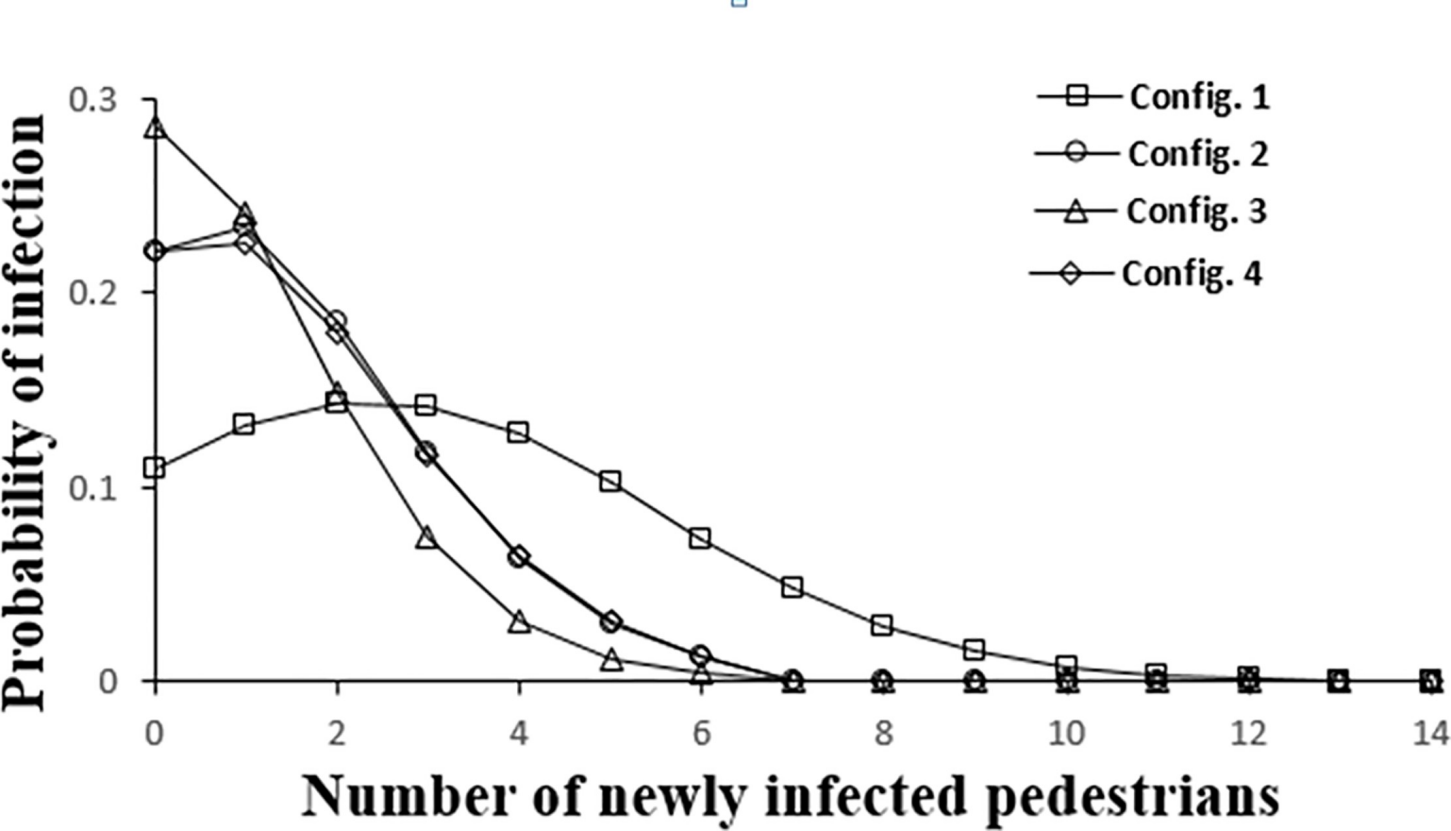
Infection distribution profile for the different configurations at P_inf_ = 0.025 and R = 1.2m with rope separation.

For a given configuration and a set of infection parameters, the mean number of newly infected pedestrians is binomially distributed to account for the demographic stochasticity in the immunity and receptivity of the susceptible population. For instance, Fig 4 represents the distribution of newly infected individuals for the four configurations at an infection probability of 0.025 and a proximate contact radius of 1.2 meters for aisles separated by ropes. While, we compute such distributions for the entire parameter space, for ease of representation in subsequent analysis, we only plot the mean of the distributions as a function of pedestrian and infection parameters.

## Results

### Rectangular floor plan

Based on our observations of common queues, we consider the situations when two pedestrians belonging to the same group can move abreast or side-by-side in the four rectangular configurations shown in Fig 3. As initial conditions, the pedestrians are distributed side-by-side inside the aisle and in front of the inlet. The spacing between the pedestrian particles is varied to differentiate between individuals of the same groups and others from different groups as mentioned earlier. As time evolves, the abreast queues turn into a single file in the exit aisles where the pedestrian speed increases (See Fig 3). We do not consider a waiting time at the exit to decrease the computational effort.

**Fig 5:**
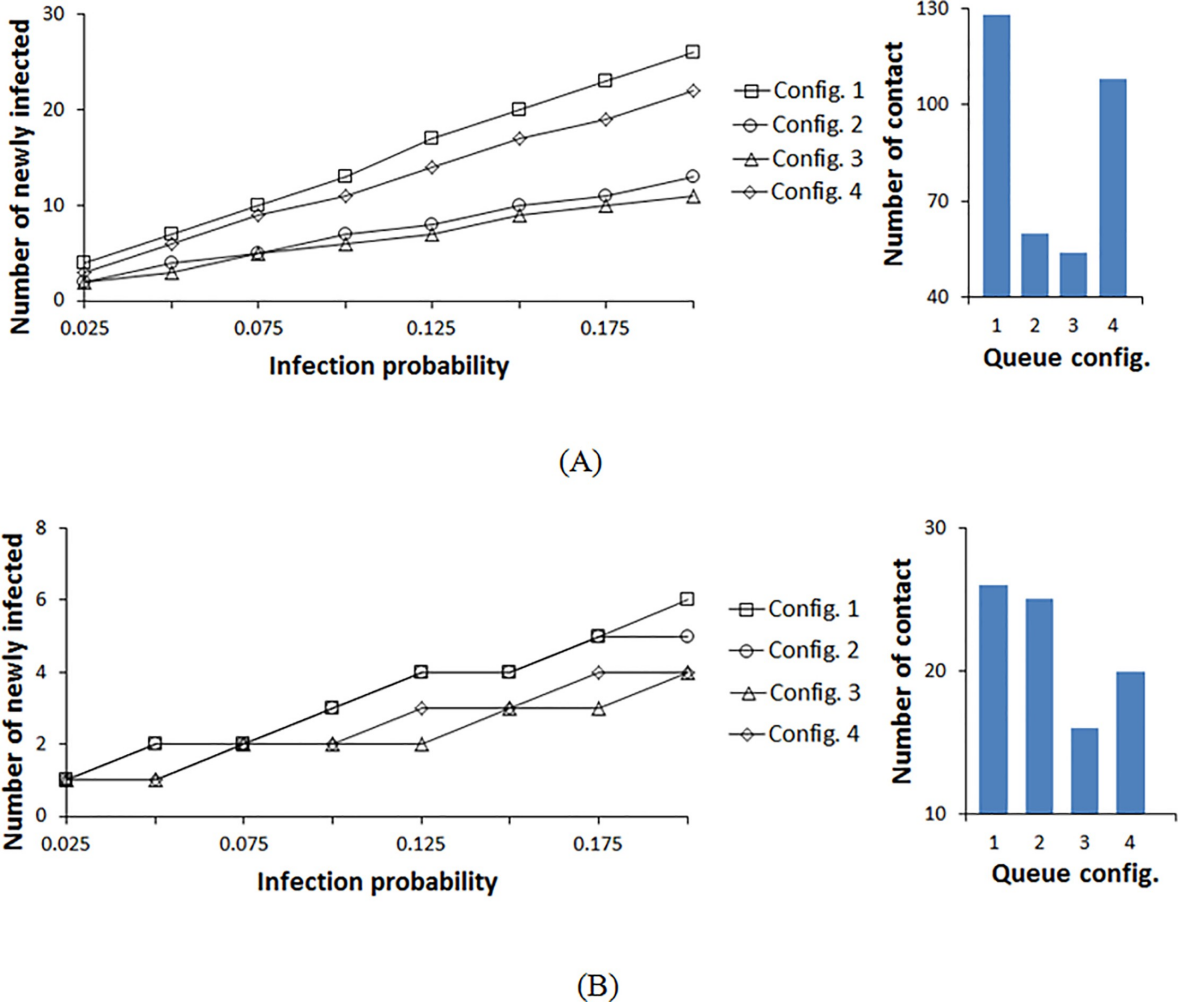
Infection distribution profile for different double queue configurations at a contact radius of 1.2m. (A) The rope is used for separation between the rows. (B) The temporary shading walls are used for separation between the rows.

With the commonly used rope separators and an infection radius less than 1.2m, which corresponds to coarse droplet mechanisms, the infective has an influence on the directly adjacent aisles on both sides. The bar chart in Fig 5A estimates the total number of contacts between the infective and the susceptible population. However, a given contact will lead to infection based on the transmission probability. Combining the contact data of the bar chart with the infection model leads to the mean distribution of infection over the probability range like in Fig 4. In Fig 5, we plot the corresponding mean of the binomial distribution for the different configurations and transmission probabilities. Configuration 3 is the best layout for all transmission probabilities, followed by configuration 2 (Fig 5A). In configuration 2, the vertical aisles are short with fewer pedestrians. Configuration 3 has the same aisle geometry as configuration 2; however, the pedestrian will exit the queue earlier (halfway) compared to that of configuration 2 which results in lower exposure time and consequently fewer contacts. Configurations 1 and 4 result in a higher mean number of infections. These configurations have long open aisles compared to configurations 2 and 3 with the lower aisle length, therefore more pedestrians are involved, and interaction occurs more frequently with pedestrians from neighboring aisles in these two configurations. Configuration 1 is the least favorable layout because diverse pedestrians from both sides come into proximity more frequently than in configuration 4 with comparatively shorter aisles. Configuration 4 is worse than configuration 2 because, at the common corners between the left and right zones, the infective comes into contact with additional pedestrians from the neighboring zones.

The use of temporary (or permanent) walls in the place of ropes limits the mixing of pedestrians within the same aisle and reduces the impact of common corners between aisles. In this case, we assume that the contagion cannot cross over to the adjacent aisles due to the solid wall barrier, therefore it results in a lower number of contacts. Fig 5B shows the mean number of infections when walls are used for crowd control. Overall, the mean number of new infections is significantly lower than when using rope separator. It can be inferred from Fig 5B that configuration 3 still results in the lowest number of infections at all transmission probabilities, and configuration 1 with long lines results in the highest number of infections in this case too. The primary difference between using rope separators and walls is for configurations 2 and 4. Configuration 2 resulted in a lower number of infections compared to 4 when using rope separators while this is reversed with walls. In configurations 3 and 4, the exit time is again shorter than that of configurations 1 and 2 resulting in lower overall contacts. Also, at 1.2m radius of infection, the configurations with long aisles and high pedestrian density corners result in higher contacts when using wall separators. This is explained by the fact that the same group of pedestrians remains in contact for a prolonged time.

**Fig 6:**
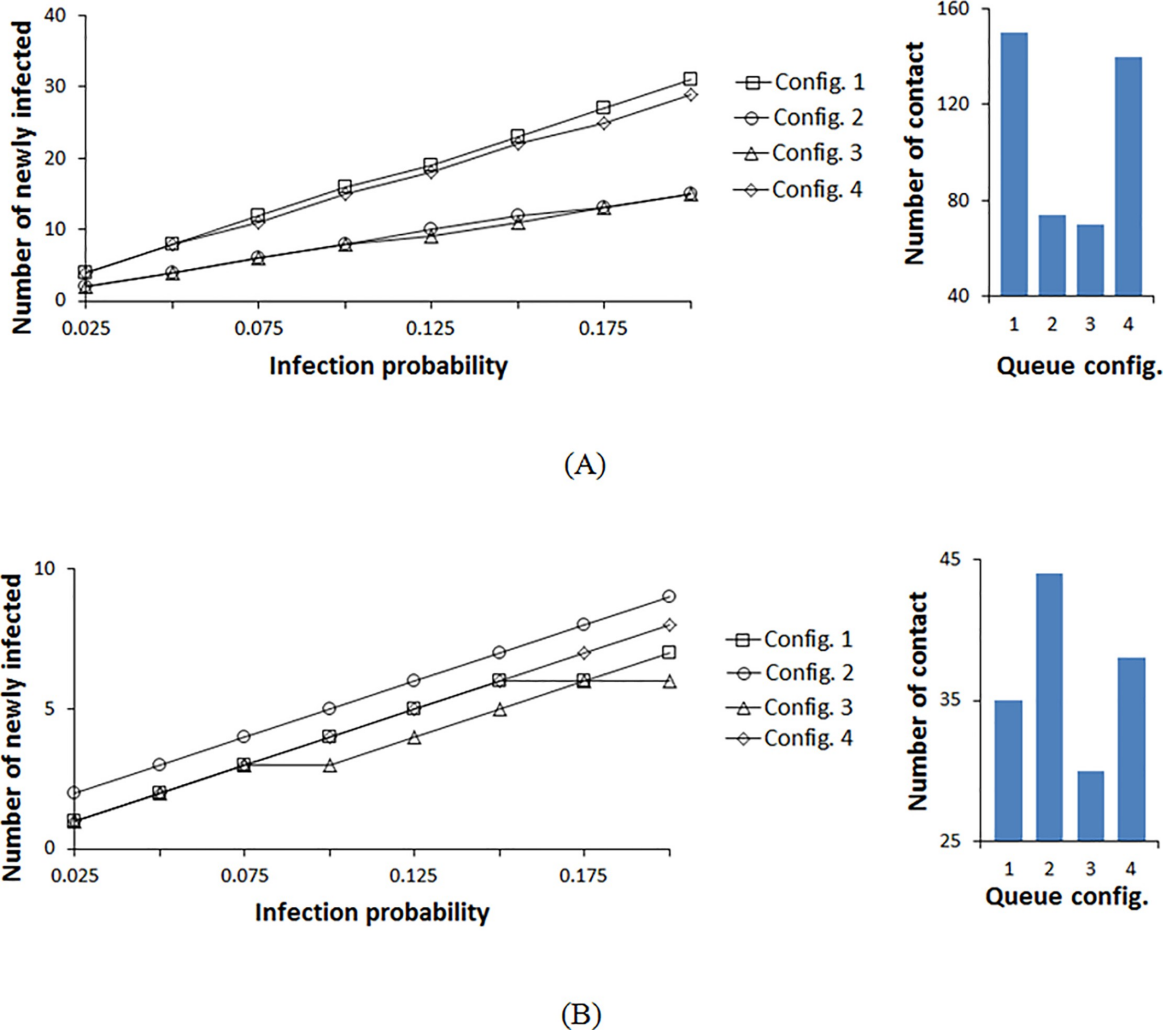
Infection distribution profile for different double queue configurations at a contact radius of 2.1 m. (A) The rope is used for separation between the rows. (B) The temporary shading walls are used for separation between the rows.

Fig 6 shows the results of repeating the transmission probability variation over the same range, but assuming the aerosol transmission mechanism with a longer contact radius of 2.1 m. Configuration 3 still results in the lowest number of contacts for both rope and wall separators. For rope separator, we observe the same pattern of results as with the lower contact radius, but with increased infection spread (Fig 6A). The differences between the configurations reduce at low transmission probabilities, therefore, the results for configurations 2 and 3, and for configurations 1 and 4 overlap. At 2.1 m contact radius, the dispersion of the fine contagion laden particles crosses the aisle boundaries to two adjacent aisles on each side. Here, the findings of configurations 2 and 3 are nearly identical since the aisles are distributed in the same manner except that configuration 3 has two separated zones. When the transmission radius expands to many neighboring aisles, pedestrians of one zone in configuration 3 come into contact not only with other pedestrians within the same zone, but with those in the adjacent zone. Accordingly, configurations 2 and 3 have the same behavior. Here, the separation of these two groups has no effective role in reducing contact. The same principle applies to configurations 1 and 4; the offset between the data of configurations 1 and 4 is reduced compared to that of the coarse droplet transmission mechanism for the same reason. Configuration 1 remains the worst layout, especially at higher probabilities, due to the elongated, abundant contact between pedestrians from adjacent aisles.

Previously, when the coarse droplet transmission with wall separator was evaluated (Fig 5B), the maximum number of contacts for configurations 1 and 2 were highest, followed by configuration 4. With aerosol transmission mechanism (R = 2.1 m) as in Fig 6B, configuration 2 remains the greatest in terms of contacts generated, followed by configuration 4, and the resultant number of contact of configuration 1 drops. At a low contact radius (R = 1.2m), pedestrian density within the circle of infection is greater in aisles than at corners. Therefore, long aisles allow greater contact time. However, an infection circle with a 2.1m radius of contact will include more pedestrians at the corners rather than the aisles. Configuration 2 has the shortest aisles, with the greatest number of corners (21 corners), which leads to a higher number of contacts.

**Fig 7:**
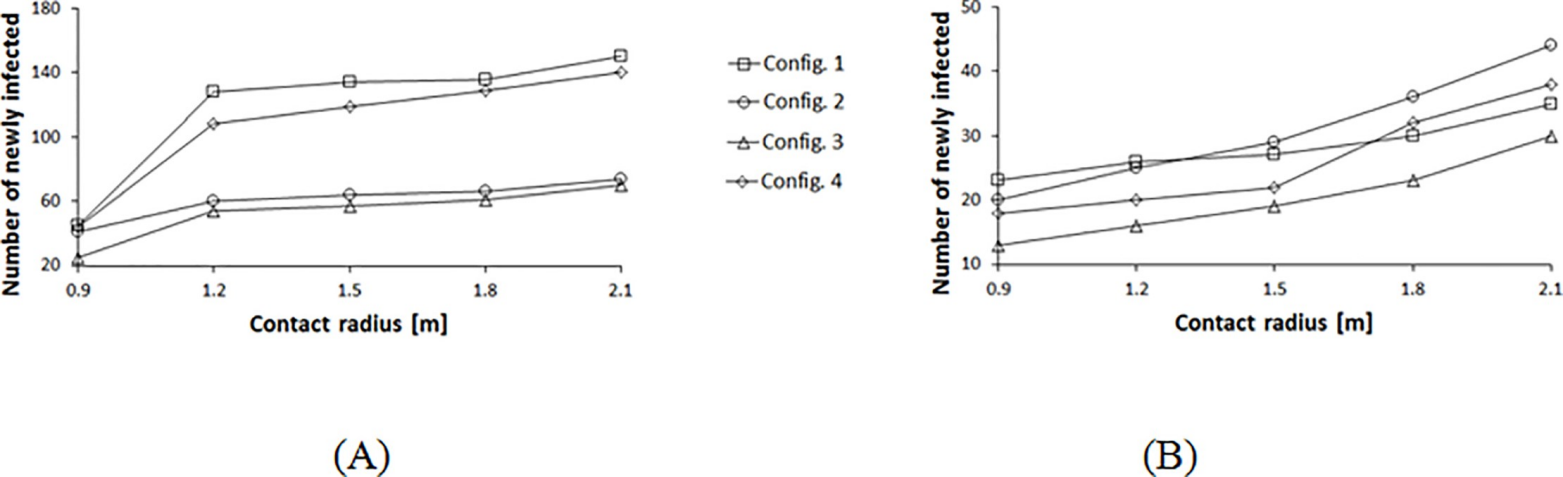
Contact distribution profile for different queue configurations with varying contact radii. (A) Rope separators used between the rows. (B) Walls used for separation between the rows.

We now explore the contacts generated between pedestrians in the four configurations assuming different infection mechanisms represented by the radius variation. Configurations 2 and 3 result in a lower number of infections for rope separators, across the range of infection radii from 0.9 to 2.1 m as shown in Fig 7A. As explained earlier, for aisles separated with ropes, shorter aisles lead to lower exposure of an infective resulting in this behavior. For walls, the combination of the radius of infection, as well as the interaction time within the aisles and at the corners alter the results as shown in Fig 7B. Each combination of infection radius and queue layout generates a different number of mean newly infected individuals. At low infection radii, short-aisle and low exit time configurations are favorable. At higher radii, configurations with less turning corners are better.

### Square floor plan

**Fig 8:**
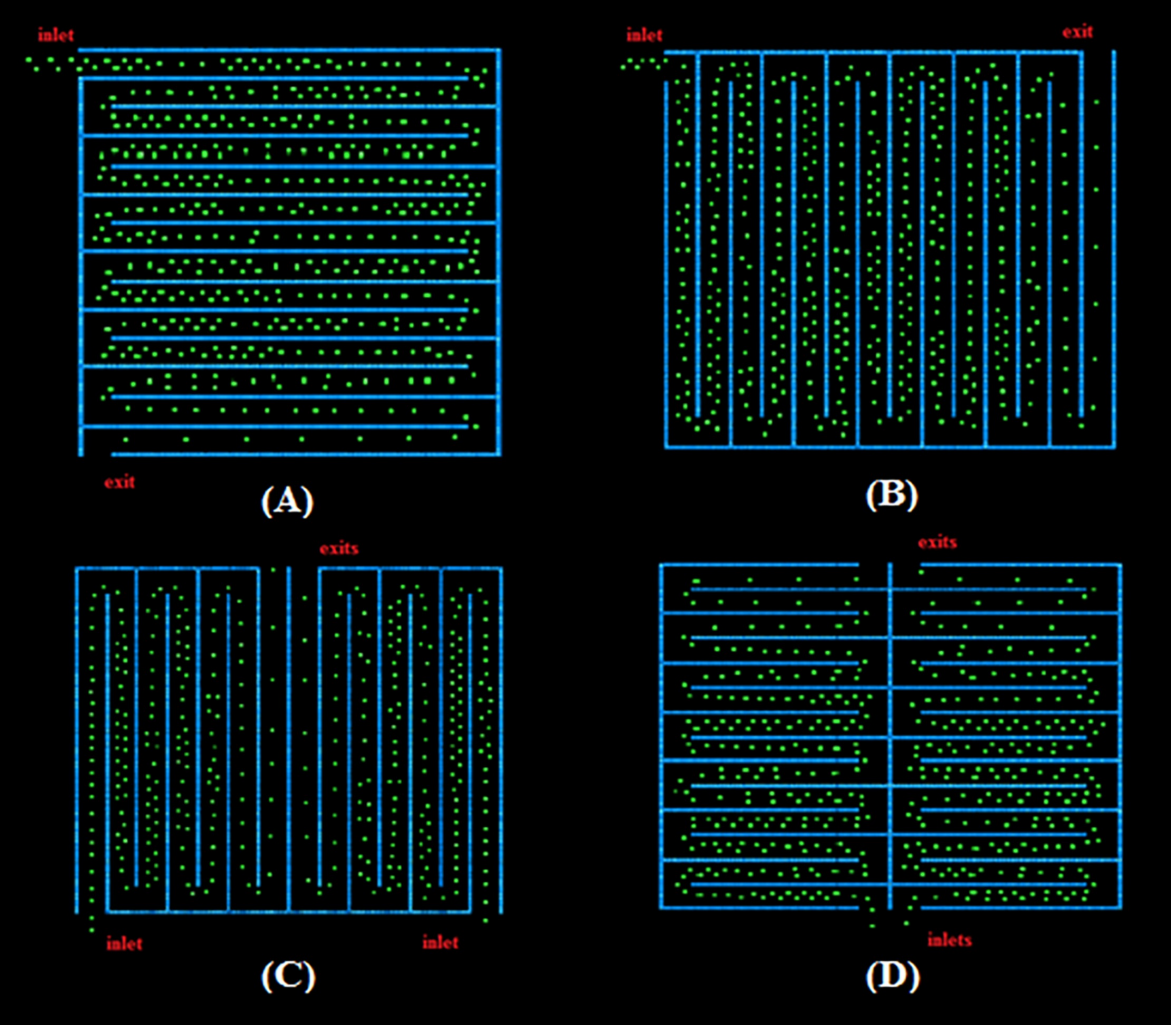
Evolution of pedestrians (t = 125s) from a simulation of abreast queue square layouts. (A) Configuration 1. (C) Configuration 2. (B) Configuration 3. (D) Configuration 4.

We now consider square layouts with the same area as the rectangular layouts discussed previously. Since the aspect ratio of the square configuration changes from that of a rectangle, the number of aisles and their dimensions vary as shown in Fig 8. Note that configurations 1 and 2 in Fig 8A and 8B, are the same except for rotation, therefore, we do not discuss them separately.

**Fig 9:**
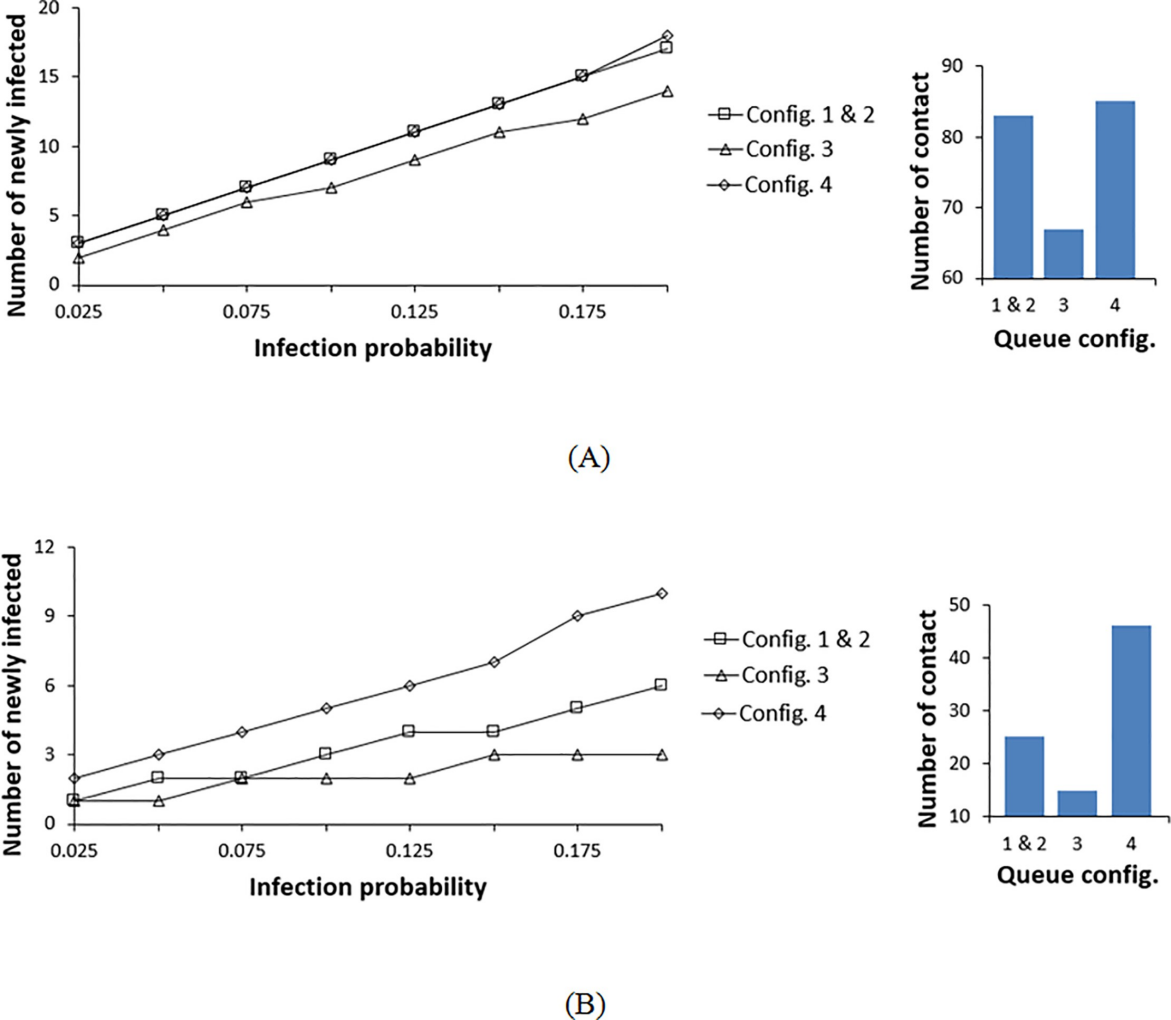
Infection distribution profile for different double queue configurations at a contact radius of 1.2 m. (A) Rope stanchions are used for separation between the rows. (B) Wall separators are used for separation between the rows.

**Fig 10:**
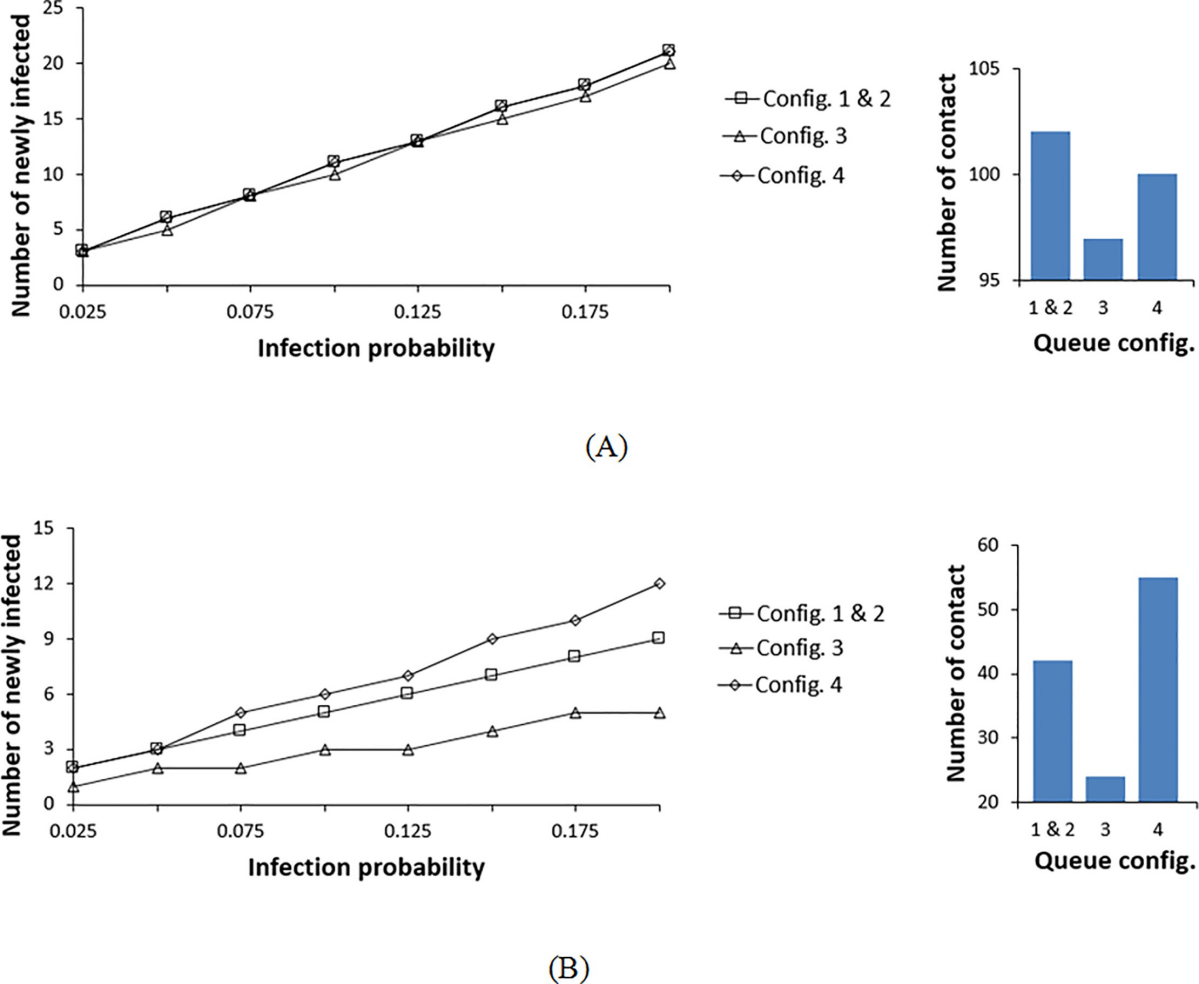
Infection distribution profile for different double queue configurations at a contact radius of 2.1 m. (A) Rope stanchions are used for separation between the rows. (B) Wall separators are used for separation between the rows.

**Fig 11:**
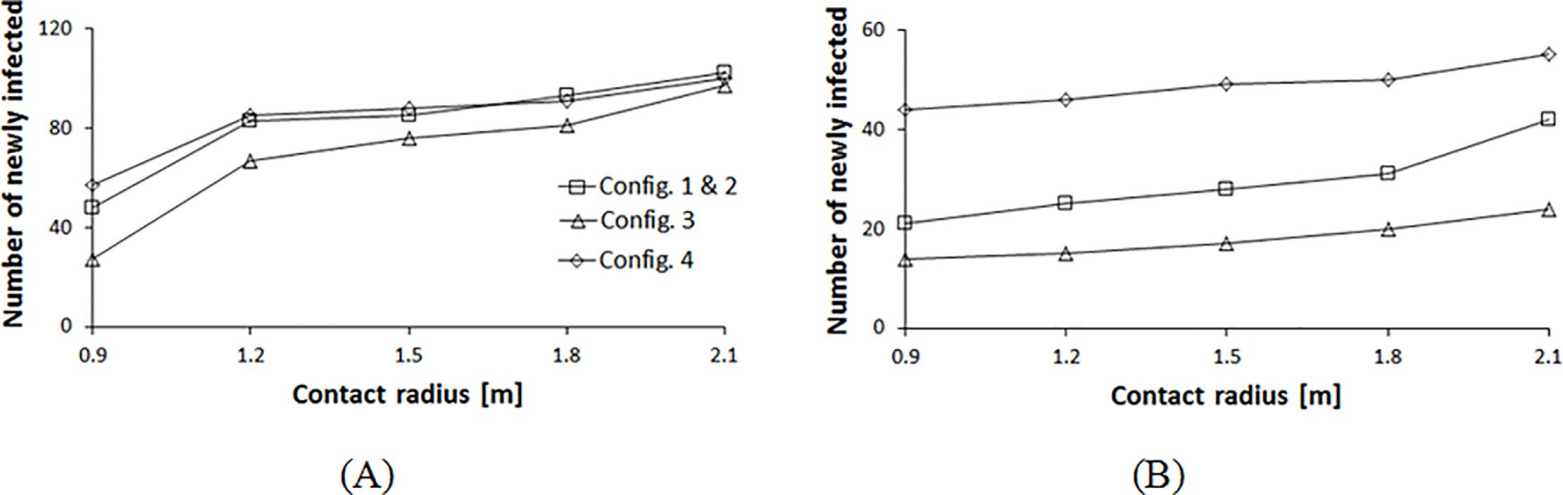
Contact distribution profile for different queue configurations with varying contact radii. (A) Rope separators used between the rows. (B) Walls used for separation between the rows.

The results shown in Figs 9–11 for these configurations are aggregate of those observed for configurations 1 and 2. Here, the best configuration is again investigated by monitoring the variation of the number of newly infected individuals in terms of infection probability and radius sweep. Looking at the four configurations, by varying the infection probability range, configuration 3 is again the most favorable, whereas, the other three configurations result in a similar number of infections when using rope separators (Fig 9A). Configuration 3 only differs from configurations 1 and 2 by the two left and right zones, enabling faster flow at the inlets and exits. In contrast to configurations 1 and 2 where pedestrians remain in the queue for a longer duration, pedestrians in configuration 3 are exiting halfway with less elapsed time in the waiting line, thus, resulting in less interaction during the shorter wait. Although configuration 4 also possesses two inlets and exits (short exit time), the number of common corners where pedestrians from both zones are at proximate contact is more than that of a rectangular layout. Also, the square configuration 4 here retains the shortest aisle length among all the configurations of the same square layout and even the rectangular ones. Although short aisles with rope separators allow less interaction as mentioned previously, shorter aisles lead to congestions at the corners where pedestrians reduce their walking speed while changing the direction of motion. Therefore, even with a shorter waiting time than the other configurations, configuration 4 allowed more frequent interactions between pedestrians of both zones resulting in a similar number of newly infected members as configurations 1 and 2, for lower contact radius (Fig 9A). Thus, the long elapsed time in the queue (aisle and corner) and the abundancy of turning corners have the same effect in increasing infection for rope separators in a rectangular floor layout. For the same configuration geometries, if the floor layout is increased, i.e. wider and longer aisles, configuration 4 will have a better performance as the interaction at the corners and in the aisles as well as the time elapsed in the queue are lower than those of configurations 1 and 2.

With temporary walls used as aisle separators, the order of the configurations alters as shown in Fig 9B. In this case, only the waiting time within the same line and congestion at the corners play an important role. Referring to Fig 8, it can be noticed that the pedestrians’ density along the aisles is almost the same between all the configurations. However, at the corners of configuration 4, pedestrians are congregated at higher density than the other layouts leading to an increase in the number of infections for Configuration 4 (Fig 9B). This is explained by the shorter aisles and the necessity to keep changing velocity direction, thus the reduction in the magnitude of the velocity components. This phenomenon also applies to the rope separator scenario. However, with ropes, the maximum interaction with pedestrians in neighboring aisles and corners is of greater importance and frequency than that within the same line. Configuration 3 remains the most favorable as it comprises a combination of moderate aisle length and less waiting time at corners.

Expanding the contact radius to 2.1 m assuming aerosol transmission mechanism, all configurations behave in the same manner for rope separators as shown in Fig 10A. Here the infective’s influence crosses multiple surrounding aisles and separation zones, therefore, the number of corners and aisles do not have any effect. For walls, the pedestrians’ distribution at the corners alters the results with minor differences (Fig 10B). Configuration 4 has the most congested corners and highest number of contacts. Fig 11 summarizes these results. At a low infection radius, for a rope separator, configuration 3 results in fewer contacts, whereas, with higher contact radii, the differences between the different configurations are reduced. For walls, pedestrians’ density at the corners leads to higher contacts for configuration 4. The short waiting time of configuration 3 makes it competitive in all conditions.

## Discussion

The modeling approach developed in this study provides a unique approach to combine pedestrian movement models and infectious disease spread models. By tracing the trajectory of each pedestrian in the time frame, the data of contacts between susceptible and infective pedestrians is obtained. Then, applying a stochastic susceptible-infected model to the contact data determines the number of newly infected individuals who are in critical contact with the infectives. This model has applications in the design of high pedestrian density locations which are often associated with infectious disease spread [2–13]. We demonstrate the approach for layout design by applying the model to various configurations of pedestrian queues and assessing the contact and infection spread dynamics as a function of various parameters.

Another aspect of the model deals with addressing the inherent uncertainty in this problem. Human movement is often guided by discretionary behaviors with respect to route and destination choices, intrinsic variability in pedestrian speed and inter-pedestrian interactions, which results in a high level of uncertainty. This aleatory uncertainty is further compounded by the combination with the infectious disease spread model, which introduces variables like transmission probability and contact radius. We parametrize the sources of uncertainty, thereby assess the conditions under which certain configurations or strategies are effective in mitigating disease spread. To account for the various transmission likelihoods and transmission mechanisms, we varied the transmission probability and contact radius in the parameter sweep. This approach can identify the effectiveness and vulnerability of a given mitigation strategy. For example, Figs 5 to 11 indicate that configuration 3 is the more effective configuration in reducing the number of contacts across different parameter combinations. Further analysis suggests that the difference between queue configurations is highest at low contact radii (e.g. 1.2 m) compared to high contact radius (2.1 m), and also for higher transmission probabilities. Such information can be useful for designing queue layouts with the objective of minimizing contact for a specific outbreak.

**Fig 12:**
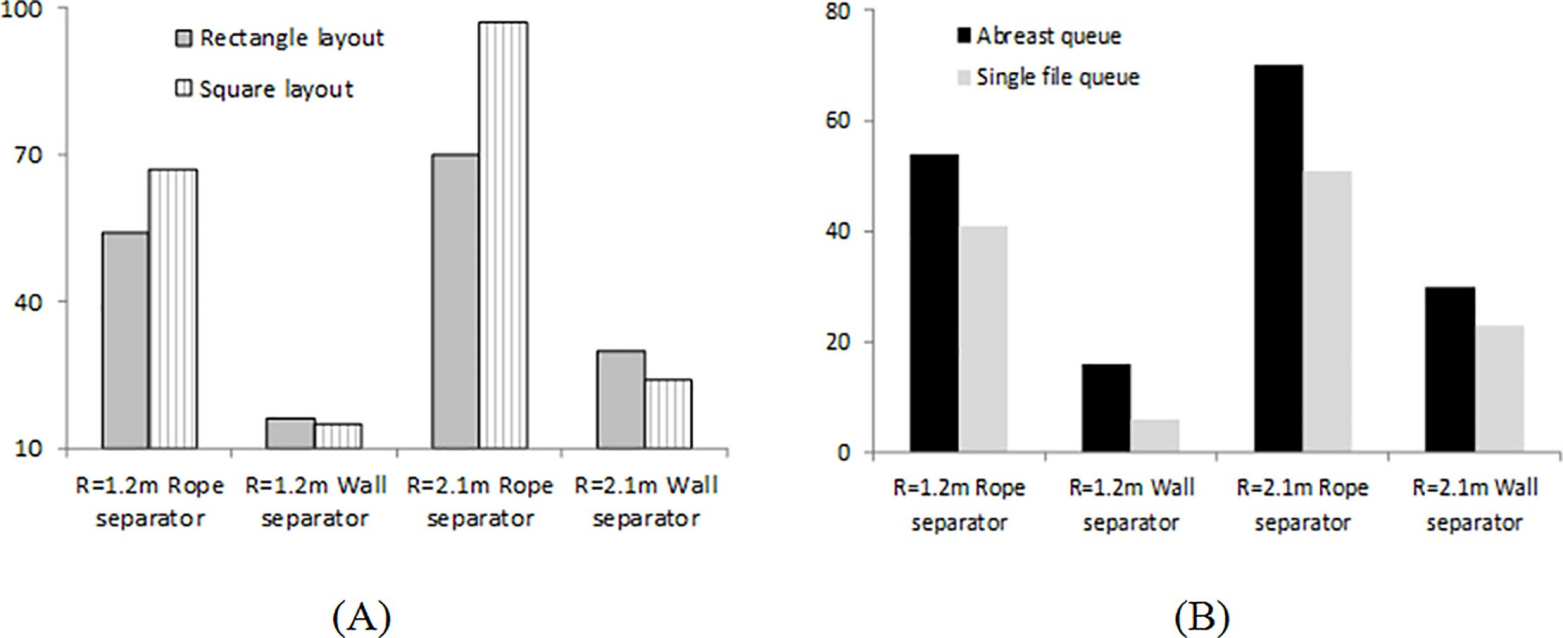
Comparison of the number of contacts between different configurations and queue arrangements. (A) Rope separators and walls for rectangular and square layout for configuration 3 with a contact radius of 1.2 m. (B) A similar comparison for the rectangular layout between the default and a single file queue setup.

We identify three main methods to reduce the number of contacts when pedestrians are waiting in queues. The shape and configuration of the layout effects the number of contacts. A longer rectangular queue with pedestrian movement aligned along the short side like in configurations 2 and 3 reduces the number of contacts. Another simple way of reducing the number of contacts in waiting queues is if temporary walls are used in place of rope separators. Such walls would potentially limit the contacts within the row, which would reduce the number of contacts from up to 55% to 75% compared to rope separators (see Fig 12A). Another approach is to reduce the aisle width to create a single file queue. Fig 12B compares the number of contacts between the default case and when single file is enforced. The overall number of contacts reduces by 8–25% in the queue configurations we considered when a single file queue is considered.

## Conclusions

There is a strong correlation between contact rates and infection rates in disease epidemics. The movement and interaction of people in high pedestrian density environments affect the number of contacts and thereby impact infectious disease spread. The mixing of susceptible and infectious individuals in these high people density environments involves pedestrian movement which is often not taken into account in the modeling studies of disease dynamics. In this paper, we developed a multiscale model for incorporating input from pedestrian dynamics models into a stochastic infection spread model. The model is applied to a ubiquitous problem of contact evolution and infectious disease spread in pedestrian waiting queues.

We evaluate the effect of queue configurations on generating contacts between neighboring pedestrians. Four distinct queues are evaluated with vertical and horizontal aisle patterns, one or two waiting zones, rectangular and square floor plans, and single-file or abreast pedestrians distributions within the control area. In these various geometrical scenarios, a comparison is made between the rope and wall separators and their effect on pedestrian interactions. With rope separators, pedestrians are allowed to interact with other pedestrians from neighboring aisles in addition to the forward and backward members in the queue within the same aisle. However, for wall separators, the interaction between pedestrians is restricted to those only within the same aisle.

We find that wall separators are very effective in reducing the number of contacts and disease spread. In some cases, replacing ropes by wall separators results in the reduction in number of contacts by more than 75%. Among the different queue configurations considered in the study, configurations with motion along short aisles lead to lower number of contacts and disease spread when rope separators are used. Also, for the same area of the queue layout, we find that rectangular configurations lead to lower number of contacts than square configurations. While the model is applied to the specific case of pedestrian queues in this paper, the general principles can be used for analysis of infectious disease spread in any high pedestrian density location.

## Supporting information

S1 File (ZIP)
